# Influence of Sex on Respiratory Syncytial Virus Genotype Infection Frequency and Nasopharyngeal Microbiome

**DOI:** 10.1128/jvi.01472-22

**Published:** 2023-02-23

**Authors:** Yi Tan, Meghan H. Shilts, Christian Rosas-Salazar, Vinita Puri, Nadia Fedorova, Rebecca A. Halpin, Siyuan Ma, Larry J. Anderson, R. Stokes Peebles, Tina V. Hartert, Suman R. Das

**Affiliations:** a Division of Infectious Diseases, Department of Medicine, Vanderbilt University Medical Center, Nashville, Tennessee, USA; b Division of Allergy, Immunology, and Pulmonary Medicine, Department of Pediatrics, Vanderbilt University Medical Center, Nashville, Tennessee, USA; c Division of Allergy, Pulmonary, and Critical Care Medicine, Department of Medicine, Vanderbilt University, Nashville, Tennessee, USA; d Division of Infectious Diseases, J. Craig Venter Institute, Rockville, Maryland, USA; e Department of Biostatistics, Vanderbilt University Medical Center, Nashville, Tennessee, USA; f Department of Pediatrics, Emory University School of Medicine, Atlanta, Georgia, USA; g Department of Otolaryngology and Head and Neck Surgery, Vanderbilt University Medical Center, Nashville, Tennessee, USA; h Department of Pathology, Microbiology and Immunology, Vanderbilt University Medical Center, Nashville, Tennessee, USA; Loyola University Chicago—Health Sciences Campus

**Keywords:** RSV, next-generation sequencing, NGS, phylogeny, genotypes, incidence, sex difference, microbiome, infants

## Abstract

Respiratory syncytial virus (RSV) has a significant health burden in children, older adults, and the immunocompromised. However, limited effort has been made to identify emergence of new RSV genotypes’ frequency of infection and how the combination of nasopharyngeal microbiome and viral genotypes impact RSV disease outcomes. In an observational cohort designed to capture the first infant RSV infection, we employed multi-omics approaches to sequence 349 RSV complete genomes and matched nasopharyngeal microbiomes, during which the 2012/2013 season was dominated by RSV-A, whereas 2013 and 2014 was dominated by RSV-B. We found non-G-72nt-duplicated RSV-A strains were more frequent in male infants (*P* = 0.02), whereas G-72nt-duplicated genotypes (which is ON1 lineage) were seen equally in both males and females. DESeq2 testing of the nasal microbiome showed Haemophilus was significantly more abundant in infants with RSV-A infection compared to infants with RSV-B infection (adjusted *P* = 0.002). In addition, the broad microbial clustering of the abundant genera was significantly associated with infant sex (*P* = 0.03). Overall, we show sex differences in infection by RSV genotype and host nasopharyngeal microbiome, suggesting an interaction between host genetics, virus genotype, and associated nasopharyngeal microbiome.

**IMPORTANCE** Respiratory syncytial virus (RSV) is one of the leading causes of lower respiratory tract infections in young children and is responsible for high hospitalization rates and morbidity in infants and the elderly. To understand how the emergence of RSV viral genotypes and viral-respiratory microbiome interactions contribute to infection frequency and severity, we utilized an observational cohort designed to capture the first infant RSV infection we employed multi-omics approaches to sequence 349 RSV complete genomes and matched nasopharyngeal microbiomes. We found non-G-72nt-duplicated RSV-A genotypes were more frequent in male infants, whereas G-72nt-duplicated RSV-A strains (ON1 lineage) were seen equally in both males and females. Microbiome analysis show Haemophilus was significantly more abundant in infants with RSV-A compared to infants with RSV-B infection and the microbial clustering of the abundant genera was associated with infant sex. Overall, we show sex differences in RSV genotype-nasopharyngeal microbiome, suggesting an interaction host genetics-virus-microbiome interaction.

## INTRODUCTION

Acute respiratory infection (ARI) due to respiratory syncytial virus (RSV) is one of the leading causes of lower respiratory tract infections (LRTIs) in young children and is responsible for high hospitalization rates and morbidity in infants and the elderly (1–5). In spite of the significant disease burden of RSV, no effective vaccine is available, partially due to the high substitution rate of the G protein and partial and transient immunity conferred from RSV infections (6–9). There are currently >60 vaccines and monoclonal antibodies in clinical trials based on a search in clinicaltrials.gov as of 20 May 2022. However, data on circulating strains that result in the greatest morbidity are necessary to reduce to RSV morbidity in infants and young children.

RSV is a negative-sense single-stranded RNA virus of the *Pneumoviridae* family (10), with a 15-kb genome (11). The two surface glycosylated proteins, G and F, mediate viral binding and subsequent membrane fusion, respectively (12, 13). There are two major RSV antigenic subgroups: RSV-A and RSV-B (14). There are spatiotemporal differences in RSV genotype distribution; for example, in a given season, different RSV lineages/genotypes cocirculate in a given location, and genotypes can be different based on geographic locations (9, 15–17). RSV (both A and B) evolve in a strongly clock-like fashion (15, 17, 18). The RSV G gene is the most variable region in both RSV-A and RSV-B genomes (15, 18). Due to its high variability, the second hypervariable portion at the C terminus of the G gene has been extensively used for RSV genotyping and sequencing (19, 20). In the past 2 decades, a sequence duplication has been found at the C-terminal ends of the G gene in both RSV-A and RSV-B (15, 21–25). The RSV-B G-gene duplication (60 nucleotide) at the C-terminal (BA-like genotype), first emerged in 1999 and has rapidly taken over the global circulation, becoming fixed by 2005; all RSV-B strains circulating since then have this duplication (21, 22). Similarly, in RSV-A, a G-gene duplication event (72 nucleotide) in the GA.2 genotype (ON1 lineage/subtype), was first reported around 2010, and it continued to spread rapidly and became the dominant strains in many countries and regions (15, 23–25). Multiple studies have noted an association between RSV genotypes and RSV disease severity (26–32). RSV-A generally causes more severe illness than RSV-B, has higher genetic variability, and is more prevalent than RSV-B (26); however, a recent study showed increased severity of the newer RSV-B genotype (BA2 with unique substitution in SH gene) (33).

In addition to viral genotypes, viral-bacterial interactions have been implicated in disease severity (34–38). Our group and others have shown that significant increases in relative abundances of certain bacterial taxa (e.g., Streptococcus, *Moraxella*, and *Haemophilus*) in the nasopharynges of infants upon RSV ARI compared to healthy infants and also correlates with a significant increase in disease severity (35, 39). Conversely, the presence of certain bacteria in the infant nose during RSV infection, such as *Lactobacillus*, can reduce the risk of subsequent wheeze (36). In addition, we have shown that the nasopharyngeal microbiome can differ between infants with RSV and human rhinovirus (37).

With the global need of an RSV vaccine, it is critical to understand how the emergence of RSV viral genotypes and virus-respiratory microbiome interactions contribute to infection frequency and severity. To understand the influence of sex and nasopharyngeal microbiome on RSV genotype infection in early life, we sequenced one of the largest collections of complete RSV genomes, which have been isolated from a population based observational birth cohort of healthy infants from middle Tennessee (the INSPIRE birth cohort), enrolled following birth during two consecutive RSV seasons, 2012 to 2014 (40), to capture the first and subsequent RSV infections in the first year of life. This cohort is unique, since we were able to catch the time-period when the G-gene 72-nucleotide duplicated RSV-A (G-72nt-duplicated RSV-A) was introduced and expanded in the population in middle Tennessee. This gave us the opportunity to not only describe the evolutionary dynamics of RSV in middle Tennessee but also to investigate the relationship between RSV genetic variation and virus-microbiome interactions on infection severity or frequency.

## RESULTS

### High-throughput sequencing of complete RSV genomes.

Our sequencing effort generated one of the largest collections of publicly available RSV complete genomes sequenced so far (349 total), including 87 sequences that were previously published (15). We attempted to sequence a total of 361 RSV RT-qPCR-positive nasopharyngeal wash samples collected during 2012 to 2014 from infants enrolled in the Infant Susceptibility to Pulmonary Infections and Asthma Following RSV Exposure (INSPIRE) cohort. We had a success rate of 92.7% in capturing complete genomes from INSPIRE samples (94.7% for both complete and partial coding sequences). In total, we acquired 207 complete RSV-A genome sequences (206 from seasons from 2012 to 2014 and one from the 2005/2006 season) and 142 RSV-B genome sequences (129 complete RSV-B genome sequences from seasons 2012 to 2014, 3 complete RSV-B genome sequences from the 2005/2006 season, 8 partial RSV-B genome sequences from seasons 2012 to 2014, and 2 partial RSV-B genome sequences from the 2005/2006 season). Together, these new complete genome sequences substantially increased the number of complete genome sequences available in GenBank.

### Diverse epidemiological dynamics of RSV in the southeastern United States from 2012 to 2014.

**FIG 1:**
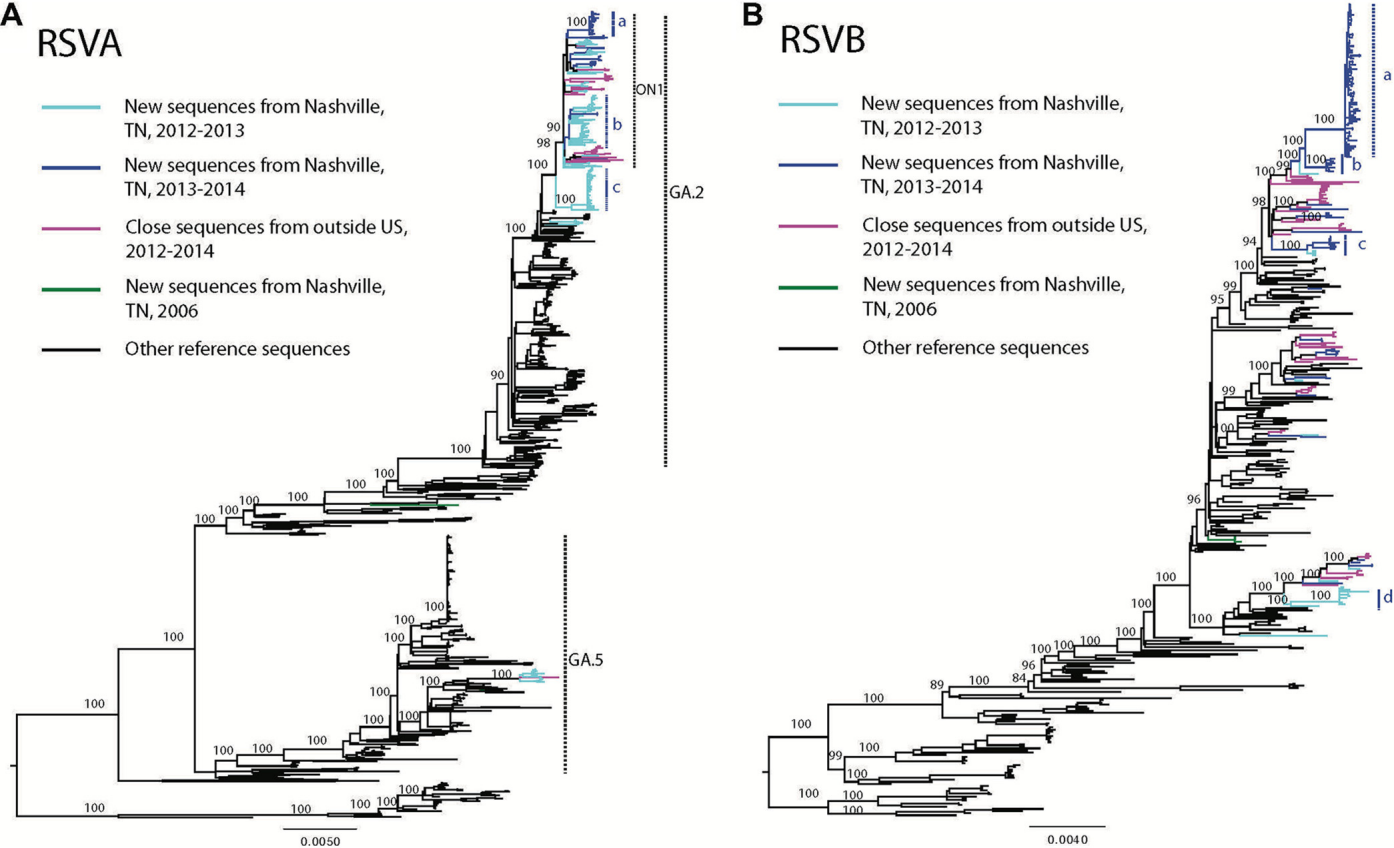
Maximum-likelihood (ML) phylogenetic trees of globally circulating RSVA (panel A) and RSVB (panel B) strains and RSV epidemics in middle Tennessee from November 2012 to March 2014. Major nodes with bootstrap value over 70% are indicated in the trees. Sequences from different places are colored and indicated in the legend keys. Clades, subclades, and middle Tennessee-specific monophyletic groups (a, b, and c in RSVA; a, b, c, and d in RSVB) are marked in the trees. The phylogenetic trees are midrooted, and the scale bars represent genetic distance.

During the main study period, November 2012 to March 2014, infants underwent surveillance during one of two RSV seasons in the middle Tennessee region of the southeastern United States: the 2012/2013 season (November 2012 to March 2013) and the 2013/2014 season (November 2013 to March 2014) (41). To gain a better understanding of the evolutionary relationships between our collection of RSV sequences in Tennessee and those circulating globally, we constructed separate maximum-likelihood (ML) phylogenetic trees of RSV-A and RSV-B of all publicly available sequences collected before or during the same study periods (Fig. 1; see also Fig. S1 and Table S1 in the supplemental material).

**FIG 2:**
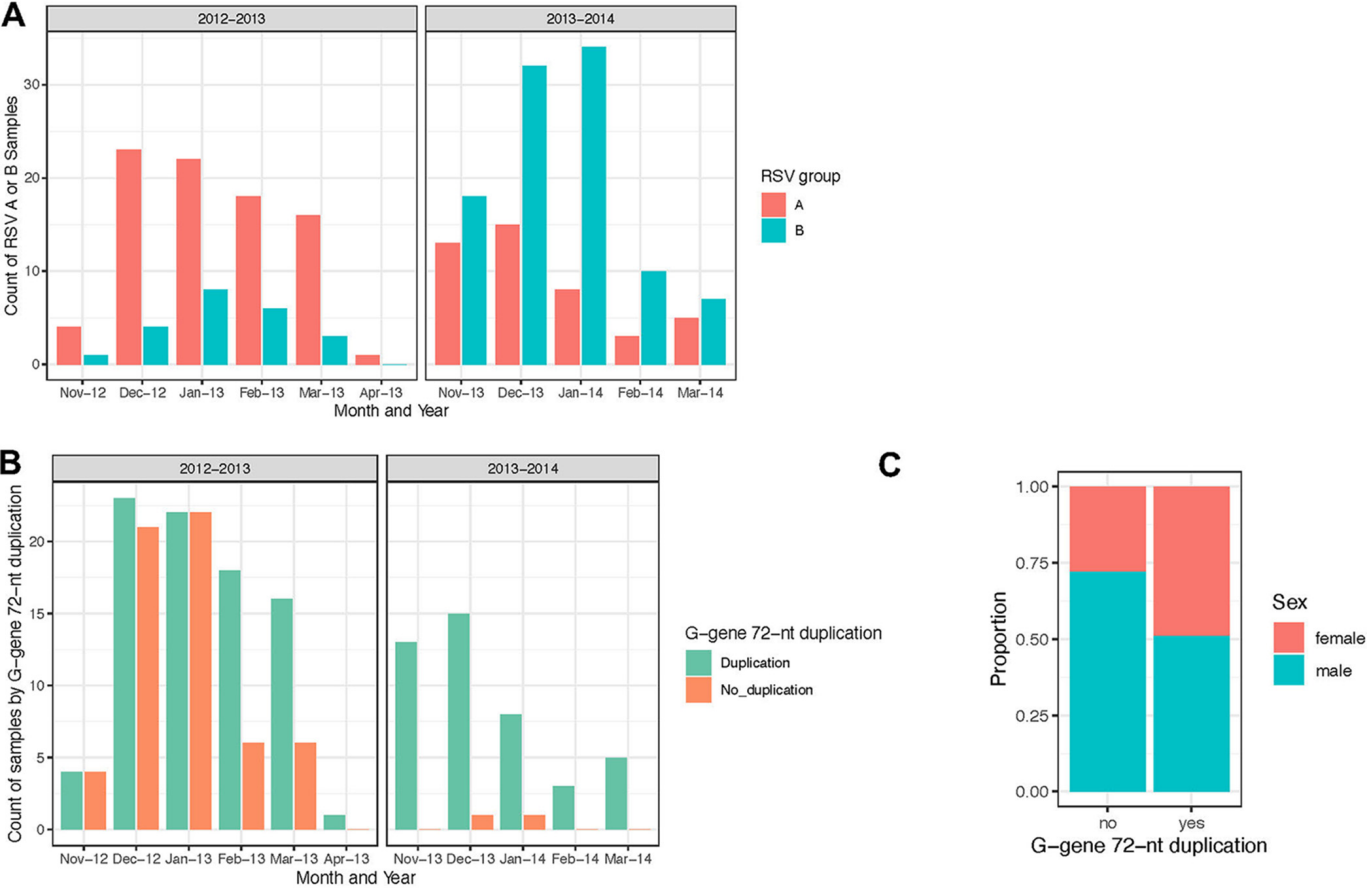
RSV-A and -B seasonality over the 2012/2013 and 2013/2014 seasons. (A) Counts of positive samples of RSV-A and RSV-B by month detected in middle Tennessee during the 2012/2013 and 2013/2014 seasons. RSV-A was the predominant strain during the 2012/2013 season, while RSV-B was the predominant strain during the 2013/2014 season. (B) Counts of, among only those with RSV-A, whether the G gene was duplicated or not by month detected in middle Tennessee during the 2012/2013 and 2013/2014 seasons. During the 2012/2013 season, both duplicated and nonduplicated G-gene RSV-A genomes were circulating. However, by the 2013/2014 season, the G-gene-nonduplicated RSV-A strains had nearly disappeared from the sampled population. (C) Among infants with RSV-A, the proportions of males and females is shown by presence or absence of the G-gene duplication. When the G-gene duplication was present, samples were evenly split between males and females. However, when the G-gene duplication was absent, males made up the majority of the samples.

While RSV-A and RSV-B cocirculated during each season in middle Tennessee, RSV-A was predominant in the 2012/2013 season, while RSV-B dominated in the 2013/2014 season (Fig. 2A). The phylogenetic analysis suggests multiple RSV-A and RSV-B genotypes/lineages cocirculated in middle Tennessee during the 2012 to 2014 seasons, with little evidence of local persistence (Fig. 1; see also Fig. S1). The ML phylogeny of global RSV-A strains suggests significant genetic diversity, especially in the two major clades, GA.2 and GA.5, in line with the genotype classification of RSV-A defined by Bose et al. (42). Most of our newly sequenced RSV-A strains fell into the GA.2 clade—both the ON1 and non-ON1 subclades—and some were in the GA.5 clade. However, the global strains isolated during the same period, 2012-2014/2015 (e.g., from New Zealand, Jordan, and Peru, labeled in magenta, Fig. 1A), were all in the GA.2 clade, specifically in the ON1 subclade, which has been identified to have a G-gene 72-nt duplication.

In middle Tennessee, we found differing epidemiological dynamics of RSV-A in both seasons (Fig. 1A). In the 2012/2013 season, GA.2 and GA.5 strains cocirculated and GA.2 strains were predominant. In particular, we were able to catch RSV-A strains without a G-gene 72-nt duplication (65 [42%] of 156) and RSV-A strains with a G-gene 72-nt duplication (91 [58%] of 156). In addition, while the majority of RSV-A were non-ON1 during the 2012/2013 season, the 2013/2014 season was mostly dominated by the ON1 genotype (Fig. 2B). In the GA.2 clade in the RSV-A phylogenetic tree, most of the middle Tennessee strains without the G-gene 72-nt duplication formed monophyletic group c and some middle Tennessee strains with the G-gene 72-nt duplication were in monophyletic group b (Fig. 1; see also Fig. S1). The rest of the sequences were spread throughout the tree. In the 2013/2014 season, all 50 sequences were in the ON1 subclade with the G-gene 72-nt duplication, and most of them formed monophyletic group a (Fig. 1; see also Fig. S1). The short internal branches of monophyletic groups a and c suggest these dominant strains were introduced into the population and spread fast in a short period of time. Monophyletic group b in ON1, with relatively longer internal branches, indicates that they have been circulating for some time prior to being detected or sampled. In addition, the middle Tennessee G-gene 72-nt duplication RSV-A strains from the two seasons did not cluster together, which suggests different RSV-A lineages circulated in two seasons with little evidence of local persistence. The historical strain collected in 2006 in middle Tennessee was neither in the GA.5 or GA.2 clade (labeled by green in Fig. 1A).

In the RSV-B global phylogenetic tree (Fig. 1B), we found significant genetic diversity of the viruses as well and the long internal branches suggest the viruses have been circulating for an even longer time before being sampled. RSV-B strains from middle Tennessee spread throughout the tree and were close to the sequences from New Zealand, Jordan, and Peru from 2012 to 2015. Particularly in RSV-B strains in middle Tennessee, sequences from the 2012/2013 season formed a small monophyletic group d, and others were spread throughout the tree. In the 2013/2014 season, although the majority of strains formed monophyletic group a, other strains formed small monophyletic groups (b and c) (Fig. 1; see also Fig. S1). Again, little evidence of local persistence of RSV-B strains was found. The historical strain collected in 2005 in middle Tennessee was phylogenetically distinct from other middle Tennessee strains collected from 2012 to 2014 (Fig. 1B).

### Association of host factors with viral genotypes.

Metadata were available for 189 RSV-A and 123 RSV-B samples from middle Tennessee. The lineage-specific demographic and clinical features of middle Tennessee RSV patients are shown in Table 1. When comparing RSV-A with RSV-B, we found that there was no significant association between RSV genotype and sex, race, mode of delivery, mode of feeding, age, type of insurance, exposure to early life antibiotics, daycare enrollment, the presence of a young sibling in the household, and exposure to household cigarette smoke. Further analysis with logistic regression modeling confirmed there were no significant associations between viral genotype and the tested demographic variables (all *P* values were >0.1).

**TABLE 1:** Baseline and clinical characteristics of infants in the INSPIRE cohort based on viral type (*n* = 312)

Characteristic	No. (%) or median (IQR)^*a*^						
	All strains				RSV-A only^*b*^		
	All (*n* = 312)	RSV-A (*n* = 189)	RSV-B (*n* = 123)	*P* ^*c*^	No duplication (*n* = 61)	Duplication (*n* = 128)	*P*
Baseline characteristics							
Female	139 (45)	80 (42)	59 (48)	0.39	18 (30)^*e*^	62 (48)	0.02
Race				0.56			0.70
Black, non-Hispanic	46 (15)	26 (14)	20 (16)		6 (10)	20 (16)	
Hispanic	26 (8)	19 (10)	7 (6)		7 (11)	12 (9)	
White, non-Hispanic	218 (70)	131 (69)	87 (71)		43 (71)	88 (69)	
Other^*e*^	22 (7)	13 (7)	9 (7)		5 (8)	8 (6)	
Birth by cesarean section	102 (33)	67 (35)	35 (28)	0.24	27 (44)	40 (31)	0.11
Any breastfeeding	236 (76)	144 (76)	92 (75)	0.89	52 (85)	92 (72)	0.07
Age (days)	148 (90–186)	153 (94–188)	138 (76–180)	0.16	160 (94–195)	149 (93–186)	0.54
Insurance type				0.40			0.26
Public	151 (48)	86 (46)	65 (53)		24 (39)	62 (48)	
Private	157 (50)	100 (53)	57 (46)		35 (57)	65 (51)	
Unknown	4 (1)	3 (2)	1 (1)		2 (3)	1 (1)	
Exposed to antibiotics	166 (53)	98 (52)	68 (55)	0.58	30 (49)	68 (53)	0.73
Enrolled in daycare	131 (42)	76 (40)	55 (45)	0.43	29 (48)	47 (37)	0.09
Had a young sibling in household	183 (59)	116 (61)	67 (54)	0.31	34 (56)	82 (64)	0.35
Exposed to household cigarette smoke	71 (23)	42 (22)	29 (24)	0.86	8 (13)	34 (27)	0.06
Clinical characteristics							
Had an LRTI	151 (48)	100 (53)^*d*^	51 (41)	0.06	28 (46)	72 (56)	0.24
Hospitalized	45 (14)	33 (17)^*d*^	12 (10)	0.09	7 (11)	26 (20)	0.20
Respiratory severity score (IQR)	3 (2–4)	3 (2–4)	3 (2–4)	0.65	2 (2–4)	3 (2–4)	0.30

a The data are presented either as the “median (IQR)” for continuous variables or as the “number (%)” for categorical variables.

b Since all circulating RSV-B genomes have a duplication in the G gene, this comparison was only performed in RSV-A samples.

c *P* values for the comparison between infants with RSV-A and RSV-B were calculated using a Wilcoxon rank sum test with continuity correction or Pearson’s chi-squared test with continuity correction, as appropriate.

d *P* values for the comparison between infants infected with a duplicated or nonduplicated RSV-A genome were calculated using a Wilcoxon rank sum test with continuity correction or Pearson’s chi-squared test with continuity correction, as appropriate.

e “Other” includes subjects of mixed race or whose race was not determined.

Further, we tested for associations between demographic/clinical features with the presence of the G-gene 72-nt duplication in RSV-A with a Wilcoxon rank sum test with continuity correction or Pearson’s chi-squared test, as appropriate. We found no significant difference in race, mode of feeding, delivery mode, age, presence of LRTI or upper respiratory tract infection (URI), and ordinal disease severity score by the presence of the G-gene 72-nt duplication. To obtain a more quantitative measure of the possible association between demographic and clinical features and virus genetic variation (the presence of the G-gene 72-nt duplication in RSV-A), we performed Bayesian tip association significance testing (BaTS) analysis using two phylogeny-trait association statistics: association index (AI) and parsimony score (PS). The results suggested that middle Tennessee RSV-A viruses do not cluster by demographic or clinical features (AI and PS both >0.05; data not shown).

However, within our middle Tennessee cohort, female infants (*P* = 0.02) were more likely to be infected with the G-gene 72-nt duplication group (Fig. 2C). Since infant boys have previously been found to have more severe RSV disease than girls (43, 44), we examined whether there were any associations between RSV-A genotype, sex, and disease severity within the INSPIRE cohort. However, using logistic regression with disease severity (measured as whether the infant had an LRTI or a URI) as the outcome, and male sex, the presence of the 72-nt duplication, and the interaction between sex and duplication as the predictors, this was not significant (male sex interquartile odds ratio [aOR] = 0.37, 95% confidence interval [CI] = 0.34 to 1.07, *P = *0.27; duplication aOR = 0.33, 95% CI = 0.15 to 0.71; *P = *0.25; interaction between male sex and duplication, *P* = 0.59).

To minimize the chance that our finding of the association between the G-gene 72-nt duplication and infant sex was spurious due to testing of multiple variables, we built a single logistic regression model, setting RSV-A genotype (G gene 72-nt duplication or no G-gene 72-nt duplication) as the dependent variable, and insurance type, age in days, exposure to early life antibiotics, any breastfeeding, race, daycare attendance, delivery mode, a young sibling in the household, exposure to cigarette smoke, and sex as the dependent variables. Exposure to cigarette smoke (aOR = 0.23, 95% CI = 0.07 to 0.81, *P = *0.02) and female sex (aOR = 0.33, 95% CI = 0.15 to 0.71, *P = *0.004) were the only factors significantly associated with increased risk of infection with the G-gene 72-nt duplicated RSV-A genotype after accounting for multiple comparisons.

### Overall summary of the microbiome during RSV infection in infants.

Matched microbiome profiles were available for 270 samples from children with RSV from the INSPIRE cohort. The median number (interquartile range [IQR]) of sequences after data processing for all samples was 10,454 (7,218 to 15,046). Read counts were normalized per sample, and average simple proportions were calculated; the most abundant genera were *Moraxella* (38.66%), Streptococcus (26.90%), Haemophilus (10.80%), *Corynebacterium* (5.28%), *Dolosigranulum* (3.91%), and Staphylococcus (1.92%). After we calculated the alpha-diversity indices, the medians (IQRs) for each index were as follows: richness, 25.90 (17.75 to 41.32); Shannon, 3.67 (2.41 to 5.80); and Simpson, 2.26 (1.61 to 3.56).

### Microbiome associations with RSV types.

Furthermore, we investigated the association between RSV types and the microbiome. RSV types data and matched microbiome profiles were available for 270 samples from the INSPIRE cohort (RSV-A, *n* = 168; RSV-B, *n* = 102). The median (IQR) number of sequences after data processing for RSV-A samples was 10,623 (7,611 to 15,688), and that for RSV-B samples was 10,056 (6,209 to 13,616). There was not a significant difference in sequencing depth by RSV type (Mann-Whitney U test, *P* = 0.08).

**FIG 3:**
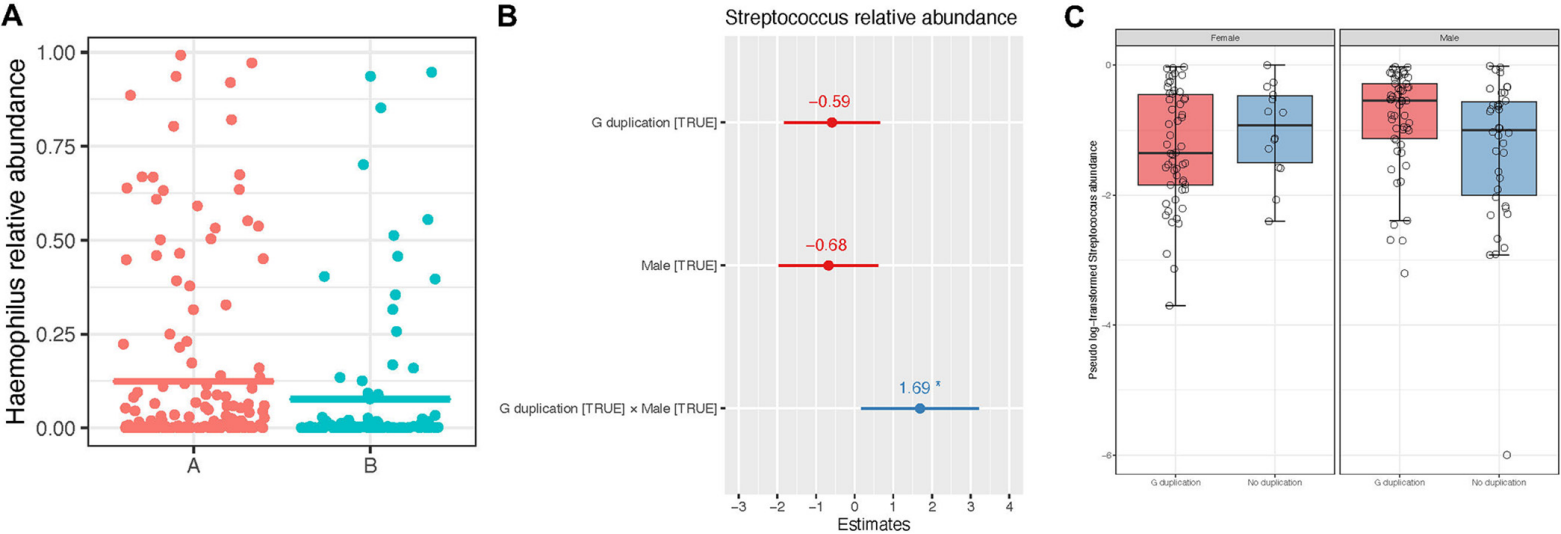
(A) Haemophilus relative abundance in children with either RSV-A or RSV-B. Each dot represents Haemophilus relative abundance in one child, while the solid line represents the mean. Children with RSV-A had a significantly higher abundance of Haemophilus compared to those with RSV-B. (B) Plot of generalized linear modeling results when the pseudo-log-transformed relative abundance of Streptococcus was set as the response variable, and male sex, presence of the 72-nt G-gene duplication, and the interaction between sex and genotype were set as the factors. The effect size estimate is shown by the dots and the 95% confidence intervals by the whiskers; asterisks indicate significance (*P* < 0.05). Estimates greater than 0 are associated with increased abundance of Streptococcus. Only the interaction of male sex and the presence of the 72-nt G-gene duplication were significantly associated with *Streptococcus* abundance; sex and/or genotype alone were not associated with Streptococcus abundance. (C) Pseudo-log-transformed relative abundance of Streptococcus (calculated as simple proportions) in RSV-A-positive infants is shown by both infant sex and the presence or absence of G-gene duplication. Each box represents the median and interquartile range, and individual points are shown as open circles. Males with the G-gene duplication had the highest relative abundance of Streptococcus.

Overall, the nasal microbiome community was similar between patients with RSV-A or RSV-B. Microbial community composition, as assessed with Bray-Curtis dissimilarities, was similar between patients with RSV-A and RSV-B (permutational analysis of variance [PERMANOVA], *R*^2^ = 0.004, *P* = 0.27), and group dispersions were also not different (*P* = 0.62). Alpha-diversity measurements were similar between infants with either RSV-A and RSV-B: the median (IQR) richness values were 26.48 (18.16 to 44.51) for RSV-A and 25.02 (17.47 to 36.73) for RSV-B (*P* = 0.06), the Shannon measurements were 3.81 (2.53 to 5.98) for RSV-A and 3.33 (2.35 to 5.40) for RSV-B (*P* = 0.26), and the Simpson measurements were 2.31 (1.70 to 3.69) for RSV-A and 2.22 (1.50 to 3.38) for RSV-B (*P* = 0.38). However, while the overall microbial community composition was similar in infants with either RSV-A or RSV-B, with the DESeq2 test, we found that Haemophilus was significantly more abundant in infants with RSV-A compared to infants with RSV-B (log_2_-fold change = −2.12, *q* = 0.002; Fig. 3A).

### Microbiome associations with the RSV-A G-gene 72-nt duplication and the effect of interactions between host sex and duplication on the microbiome.

Similarly, among only infants with RSV-A, the overall nasal microbiome community was comparable between those with or without the G-gene 72-nt duplication. Microbial community composition, as assessed with Bray-Curtis dissimilarities, was similar regardless of the presence of the G-gene 72-nt duplication (*R*^2^ = 0.007, *P* = 0.29), and group dispersions were also not significantly different (*P* = 0.76). Alpha-diversity measurements were similar between those with or without the G-gene 72-nt duplication and were not significantly different. The median (IQR) richness was 28.71 (18.40 to 47.96) with the duplication and 23.55 (17.39 to 41.42) without the duplication (*P* = 0.36), the Shannon measurements were 3.98 (2.98 to 6.02) for duplication and 2.92 (2.11 to 5.57) for no duplication (*P* = 0.245), and the Simpson measurements were 2.58 (1.88 to 3.77) for duplication and 2.11 (1.39 to 3.05) for no duplication (*P* = 0.242).

As we observed a difference in sex distribution depending on whether there was a 72-nt duplication in the G gene (Fig. 2C), we explicitly examined associations between host sex, RSV genotype, and the microbiome with generalized linear modeling. Taxonomic (genera) and community-level (alpha-diversity, top principal coordinates) features of the microbiome were set as the response variables, with RSV genotype, host sex, and the genotype/sex interaction factor as the predictor variables. With this testing, Streptococcus was the only genus significantly associated with the interaction between host sex and RSV genotype (estimate [standard error] 1.69 [0.77]; *P* = 0.03); in this model, neither male sex (estimate [standard error] −0.68 [0.66]; *P* = 0.31) nor presence of the 72-nt G-gene duplication (estimate [standard error] −0.59 [0.63]; *P* = 0.35) alone was significantly associated with Streptococcus abundance (Fig. 3B). Overall, males with the 72-nt G-gene duplication had the highest relative abundance of Streptococcus compared to all groups (Fig. 3C). The interaction between host sex and RSV genotype and its association with Streptococcus abundance can be seen in Fig. 3C: males with the 72-nt G-gene duplication had a higher relative abundance of Streptococcus compared to males without the duplication; in females, this trend was reversed. Among all of the tested indices of microbial richness or diversity, none were significantly associated with the interaction of host sex and genotype.

## DISCUSSION

Our study described the epidemiological dynamics of RSV epidemics in middle Tennessee, during 2012 to 2014, the key period when the evolutionary event of the G-gene 72-nt duplication in the RSV-A genome was introduced into the population. For the first time, it allowed us to investigate the association between RSV genetic variation with host characteristics, systematically. We revealed two differing RSV epidemics in middle Tennessee. The middle Tennessee RSV 2012/2013 epidemic season (November 2012 to March 2013) was dominated by RSV-A strains and was characterized by multiple introductions and rapid spread of both G-72nt-duplicated and non-G-72nt-duplicated RSV-A strains, while in the 2013/2014 epidemic season (November 2013 to March 2014), RSV-B strains were predominant and cocirculated with G-72nt-duplicated RSV-A strains. Both RSV-A and RSV-B strains showed little evidence of local persistence in two seasons in middle Tennessee. We also found that RSV-A strains were associated with more severe clinical symptoms and significantly higher nasopharyngeal abundance of Haemophilus (45, 46). Within the INSPIRE cohort, RSV-A, non-G-72nt-duplication strains were more frequent during infection in males than females; however, strains with the G-72nt duplication infected males and females at similar rates. We noted at interaction between RSV genotype and sex influenced the microbiome, since males with G-72nt-duplication strains tended had higher abundances of Streptococcus, while neither sex nor RSV genotype alone were associated with Streptococcus abundance.

Like influenza, RSV epidemics show significant seasonality and typically peak in the winter in temperate regions, during October to March in the Northern Hemisphere (47–49), and during May to August in the Southern Hemisphere (50, 51). There is limited RSV seasonality in tropical regions; however, RSV activity is associated with the rainy season in tropical climates (52–54). RSV not only shares the similar seasonality pattern as influenza but also presents a similar alternating pattern of dominant viral subtypes or lineages (47, 55). In line with these findings, our study of two RSV seasons in middle Tennessee showed strong annual seasonality with peaks in December and/or January, and RSV-A and RSV-B rotated to take the predominant role in two consecutive seasons in middle Tennessee (Fig. 2A). Studies such as this one, which elucidates the RSV epidemiological dynamics are needed to develop provide important information in developing an effective global RSV vaccine and prevention strategy, since the optimal vaccine strategy not only depends on the timing of RSV activity but also on the predominant strain of RSV circulating during each season.

Our phylogenetic analyses of global RSV-A and RSV-B strains suggest that there were complex epidemiological dynamics of RSV epidemics in middle Tennessee, during the 2012/2013 and 2013/2014 seasons. Multiple RSV-A and RSV-B strains were cocirculated in the population in both seasons. Specifically, equivalent numbers of RSV-A non-G-72nt-duplication, RSV-A G-72nt-duplication, and RSV-B strains cocirculated during the 2012/2013 season, while RSV-B (all with G-60nt-duplicated) strains were dominant and non-G-72nt-duplicated RSV-A strains had mostly disappeared during the 2013/2014 season. Little local persistence of RSVA G-72nt-duplication strains and RSV-B strains over two seasons also suggests that the predominant strains in 2013/2014 season could be new viral introductions from outside middle Tennessee. RSV-A G-72nt-duplication strains (ON1 lineage) have been detected and reported in different parts of the world since 2010 (17, 56–60). In our study, the disappearance of RSV-A strains without G-72nt duplication in 2013/2014 might suggest that RSV-A G-72nt-duplication strains or ON1 lineage entered the population and replaced non-G-72nt-duplication strains in 2012. However, because we did not have the data for the previous 2011/2012 season, we could not say whether the G-72nt duplication or ON1 lineage was first introduced to the population in 2012. ON1 strains might have been circulating for a while before we detected it in 2012. Similarly, we are not sure whether RSV-A non-G-72nt-duplication strains would come back to the population after 2014 because of the lack of viral data in the 2014/2015 season.

A multitude of studies have shown sex differences in the incidence and severity of respiratory tract infections (44, 61, 62). In the case of seasonal influenza virus, studies show that typically prepubertal males, compared to age-matched females, required hospitalization more often and have more severe infections (5–7). Similarly, in RSV infections, boys were found to be more severely affected than girls (43, 44). This is the first study to our knowledge to show a sex-based difference in frequency of viral infection of an RSV genotype, where c-terminal non-G-72nt-duplicated (GA2 non-ON1 or GA5) RSV-A genotypes infected male infants more commonly than females. However, upon acquisition of the duplication, the ON1 lineage/genotype was found to infect both male and female infants with equal frequency. Without further studies, it would be purely speculative that this sex difference in frequency of infection is a gain of function or a loss of function. However, previously in the context of RSV-B, our team has experimentally shown that with the acquisition of a C-terminal 60-nt duplication (in the same region of the G gene), there was a fitness advantage, by increasing viral attachment, compared to the virus without the duplication (63). Our global sequence analyses of RSV-A G gene with or without duplication showed no significant differences by infant sex (see Table S2). However, this lack of difference in infectivity in the global data set could be attributed to biased samples (mostly coming from hospitalized cases), samples from all age groups, and prior unknown history of RSV infection. In contrast, our observational cohort was specifically designed to capture early life (first) infections in infants and the study design was population based, not biased toward only hospitalized cases, as all cases of RSV severity were included.

Another important host factor for infectivity is the nasal microbiome. Our comparison of RSV-A versus RSV-B shows a trend of RSV-A patients with more severe clinical symptoms than RSV-B patients in middle Tennessee, trending toward more LRTIs and more hospitalizations, which is consistent with previous studies (26–32). In addition, our study found that RSV-A strains were associated with higher relative abundance of nasopharyngeal Haemophilus. High abundance of Haemophilus in the noses of infants infected with RSV has previously been associated with greater disease severity and a local massive antiviral cytokine response (45, 46, 64).

Our study has several strengths, including the unique INSPIRE observational cohort which allowed us to investigate the comparison of G-72nt duplication versus non-G-72nt duplication in RSV-A, where we discovered a sex bias of infectivity. We observed that males with G-72nt-duplication RSV-A tended to have higher abundances of Streptococcus; Streptococcus abundance was not associated with either sex or RSV genotype alone, suggesting host and viral factors may interact to influence the microbiome. Streptococcus in the nose of RSV-infected children has previously been associated with more severe disease (39, 46); although males with the G-gene 72-nt duplication did not have significantly more severe disease in this cohort, future studies examining interactions between the microbiome, RSV genotype, and host demographics and their influence of disease severity would be of interest.

However, we also acknowledge several limitations. Since we do not have a population-level survey of RSV sequences in middle Tennessee from before or after our study period, we do not know when the G-gene 72-nt duplication first appeared in the population, nor do we know if the G-gene 72-nt duplication has since become completely fixed in circulating RSV-A. Second, as we did not have access to another population-level cohort study of all circulating RSV strains during the same period from a different region, we were unable to validate our findings of sex-based infectivity based on RSV genotype within another cohort. Since we were unable to confirm our findings in the whole global data set, perhaps this G-gene 72-nt duplication-based sex difference was unique to middle Tennessee. Third, our microbiome profiling method relies on a short marker fragment of the 16S rRNA gene, which is often not sufficient to identify bacteria to the species level. Therefore, although we observed increased Haemophilus relative abundance in the nasopharynx of infants with RSV-A compared to RSV-B and increased Streptococcus relative abundance in the nasopharynx of male infants with G-72nt-duplicated RSV-A compared to males without the duplication and females, we do not know the species of either of these genera and thus whether they are potential pathogens or commensals.

Overall, described the epidemiological dynamics of RSV epidemics in middle Tennessee, during 2012 to 2014, the key period when the evolutionary event of the G-gene 72-nt duplication in the RSV-A genome was introduced into the population by sequencing on of the largest collection of RSV complete genomes published so far. Further, this study, for the first time, describes a sex-based difference in frequency of viral genotype infection and association of nasopharyngeal microbiome with sex and RSV genotypes. Understanding how different RSV genotypes and host factors contribute to disease severity is vital for creating an effective vaccine. This population-based study of community RSV infection provides an important evolutionary perspective on seasonal variation of community RSV infections among healthy term infants.

## MATERIALS AND METHODS

### Overview of INSPIRE cohort.

The Infant Susceptibility to Pulmonary Infections and Asthma Following RSV Exposure (INSPIRE) is a population-based birth cohort of previously healthy, term infants born between June and December of 2012 and 2013, designed so that the first RSV infection during infancy could be studied. The detailed methods for INSPIRE have been previously reported (40). Sociodemographic characteristics of each infant were obtained at enrollment. In order to capture an infant’s first RSV ARI, biweekly respiratory illness surveillance was performed during the winter viral season (November to March) of each infant’s first year of life. Infants who met prespecified criteria for an ARI had an in-person visit, which included a nasal wash for viral identification and characterization of the nasopharyngeal microbiome, assessment of the ARI severity using a respiratory severity score, and blood draw at 1 year for RSV serology (65). The Institutional Review Board of Vanderbilt University approved this study, and informed consent was obtained from the legal guardians of each infant.

### Viral RNA extraction, whole-genome sequencing, assembly, and annotation.

Methods for virus isolation, RNA extraction and cDNA synthesis, library preparation, and next-generation sequencing, and genome assembly and annotation have been previously described (15, 17). In brief, extraction of the viral RNA was performed using 140 μL of the nasal wash sample in viral transport medium using a viral RNA minikit (Qiagen, Germany). Four forward reverse transcription (RT) primers were designed, and four sets of PCR primers were manually picked from primers designed across a consensus of complete RSV genome sequences, as described previously (15). The four forward RT primers were diluted to 2 μM and pooled in equal volumes. cDNA was generated from 4 μL of undiluted RNA, using the pooled forward primers and SuperScript III Reverse Transcriptase (Thermo Fisher Scientific, Waltham, MA). Four independent PCRs were performed on 2 μL of cDNA template using either AccuPrime *Taq* DNA polymerase (Thermo Fisher Scientific) or Phusion High Fidelity DNA polymerase (New England Biolabs, Ipswich, MA) to generate four overlapping ~4-kb amplicons across the genome. Amplicons were verified on 1% agarose gels, and excess primers and deoxynucleoside triphosphates were removed by treatment with Exonuclease I (New England Biolabs) and shrimp alkaline phosphatase (Affymetrix, Santa Clara, CA) for 37°C for 60 min, followed by incubation at 72°C for 15 min. Amplicons were quantitated using a SYBR green dsDNA detection assay (SYBR green I nucleic acid gel stain; Thermo Fisher Scientific), and all four amplicons per genome were pooled in equal concentrations.

Samples were sequenced using both the MiSeq (Illumina) and Ion Torrent PGM (Thermo Fisher Scientific) to overcome platform-specific errors. The Illumina libraries were prepared using a Nextera DNA sample preparation kit (Illumina, Inc., San Diego, CA) with half reaction volumes. Briefly, 25 ng of pooled DNA amplicons was tagmented at 55°C for 5 min. Tagmented DNA was cleaned with the ZR-96 DNA Clean & Concentrator kit (Zymo Research Corporation) and eluted in 25 μL of resuspension buffer. Illumina sequencing adapters and barcodes were added to tagmented DNA via PCR amplification, where 20 μL of tagmented DNA was combined with 7.5 μL of Nextera PCR Master Mix, 2.5 μL of Nextera PCR Primer Cocktail, and 2.5 μL of each index primer (Integrated DNA Technologies, Coralville, IA) for a total volume of 35 μL per reaction. Thermocycling was performed with 5 cycles of PCR, as per the Nextera DNA sample preparation kit protocol (3 min at 72°C, denaturation for 10 s at 98°C, annealing for 30 s at 63°C, and extension for 3 min at 72°C) to create a dual-indexed library for each sample. After PCR amplification, 10 μL of each library was pooled into a 1.5-mL tube, and the pool was cleaned two times with Ampure XP reagent (Beckman Coulter, Inc.) to remove all leftover primers and small DNA fragments. The first cleaning used a 1.2× volume of the Ampure reagent, while the second cleaning used a 0.6× volume of the Ampure reagent. The cleaned pool was sequenced on the Illumina MiSeq v2 instrument (Illumina, Inc.) with 300-bp paired-end reads.

In addition to Illumina sequencing, for Ion Torrent PGM (Thermo Fisher Scientific) sequencing, 100 ng of pooled DNA amplicons were sheared for 7 min, and Ion-Torrent-compatible barcoded adapters were ligated to the sheared DNA using the Ion Xpress Plus Fragment Library kit (Thermo Fisher Scientific) to create 400-bp libraries. Libraries were pooled in equal volumes and cleaned with Ampure XP reagent (Beckman Coulter, Inc., Brea, CA). Quantitative PCR was performed on the pooled, barcoded libraries to assess the quality of the pool and to determine the template dilution factor for emulsion PCR. The pool was diluted appropriately and amplified on ion sphere particles (ISPs) during emulsion PCR on the Ion One Touch 2 instrument (Thermo Fisher Scientific). The emulsion was broken, and the pool was cleaned and enriched for template-positive ISPs on an Ion One Touch ES instrument (Thermo Fisher Scientific). Sequencing was performed on the Ion Torrent PGM using 318v2 chips (Thermo Fisher Scientific).

### RSV genome assembly and annotation.

Sequence reads were sorted by barcode, trimmed, and *de novo* assembled using CLC Bio’s *clc assembler* program, formerly known as *clc novo assembly* (http://resources.qiagenbioinformatics.com/manuals/clcgenomicsworkbench/852/index.php?manual=De_novo_assembly.html), and the resulting contigs were searched against custom, full-length RSV nucleotide databases to find the closest reference sequence. All sequence reads were then mapped to the selected reference RSV sequence using CLC Bio’s *clc_mapper_legacy*, formerly called as *clc_ref_assemble_long* program (http://resources.qiagenbioinformatics.com/manuals/clcassemblycell/current/index.php?manual=Options_clc_mapper_legacy.html). At loci where both Ion Torrent and Illumina sequence data agreed on a variation (compared with the reference sequence), the reference sequence was updated to reflect the difference. A final mapping of all next-generation sequences to the updated reference sequences was performed with CLC Bio’s *clc_mapper_legacy* program. Curated assemblies were validated and annotated with the viral annotation software Viral Genome ORF Reader, VIGOR 3.0 (66), before submission to GenBank. VIGOR was used to predict genes, perform alignments, ensure the fidelity of open reading frames, correlate nucleotide polymorphisms with amino acid changes, and detect any potential sequencing errors. The annotation was subjected to manual inspection and quality control before submission to GenBank. All 339 sequences generated as part of this study were submitted to GenBank as part of the BioProject IDs PRJNA241108, PRJNA225816, and PRJNA267583, with accession numbers KJ672424.1 to KX894807.1 (KM042382.1, KM042387.1, and KU950597.1 are partial sequences).

In total, we recovered 207 complete RSV-A genome sequences (206 from seasons 2012 to 2014 and one from the 2005/2006 season) and 142 RSV-B genome sequences (129 RSV-B complete genome sequences from seasons 2012 to 2014, 3 RSV-B complete genome sequences from the 2005/2006 season, 8 RSV-B partial genome sequences from seasons 2012 to 2014, and 2 RSV-B incomplete genome sequences from the 2005/2006 season).

### Phylogenetic analyses.

We performed phylogenetic analyses on 339 RSV complete genome sequences sequenced by us (207 RSV-A and 132 RSV-B)—this total includes 87 sequences (59 RSV-A and 28 RSV-B) which were previously published (15)—collected in the middle Tennessee region from infants in the INSPIRE cohort between 2012 and 2014, together with reference strains available on GenBank (http://www.ncbi.nlm.nih.gov/genbank/; January 2017). In total, we generated two complete genome sequences data sets: 859 RSV-A (207 new sequences and 652 from GenBank) and 392 RSV-B (132 new sequences and 260 from GenBank). Sequences of these two data sets were aligned separately using the MUSCLE program in MEGA 6.0 with manual adjustment (67). Potential recombination within these sequences was screened using seven methods (RDP, GENECONV, Chimaera, MaxChi, SiScan, 3Seq, and BootScan) implemented in the Recombination Detection Program version 4.46 (RDP4) (68). A general time reversal (GTR) nucleotide substitution model with a gamma distribution of among-site rate variation (GTR+Γ) was selected as the best-fit model by Modeltest in MEGA 6.0 and used in all tree inference methods. ML phylogenies were inferred in RAxML with 1,000 bootstrap replications (69). In addition, middle Tennessee region-only trees were inferred using the Bayesian Markov chain Monte Carlo (BMCMC) method available in MrBayes version 3.2.5 (70), run for 1 × 10^8^ steps using the same model setting. Trees were sampled every 1 × 10^4^ steps, with the first 1,000 trees discarded as burn-in.

### Association analyses between demographic and clinical features of RSV patients with genetic variation.

Genome sequences and demographic and clinical information were available for 312 (189 RSV-A and 123 RSV-B) of 349 (207 RSV-A and 142 RSV-B) infants from the middle Tennessee region as part of the INSPIRE cohort. Within this cohort, 128 of 189 infants with RSV-A strains had a G-gene duplication in their genomes. Virus sequences were also grouped by metadata information individually (categorical variables), specifically by sex (male or female), race (black non-Hispanic, Hispanic, white non-Hispanic, other), mode of delivery (vaginal or caesarean), mode of feeding (breastfeeding or formula), and respiratory tract infection (lower respiratory tract infection [LRTI] or upper respiratory tract infection [URI]). Age of the baby when the sample was taken and an ordinal respiratory disease severity score were also documented (quantitative characteristics). Comparisons of host information between RSV-A and RSV-B and the effects of G-gene duplication on demographic and clinical features were performed by the Wilcoxon rank sum test for quantitative characteristics and chi-square test for categorical variables, respectively. A *P* value of <0.05 was considered statistically significant.

To further explore the associations between viral genotype and host demographic data, we next performed logistic regression with the *lrm* function in the R package *rms* (v6.2-0) (71), setting viral genotype as the dependent variable. Two separate models were run: (i) the dependent variable set as either RSV A or RSV B (model 1) and (ii) among RSV-A only, the dependent variable was set as presence of the duplication in the G gene (model 2). For all models, age, sex, race/ethnicity, delivery mode (vaginal or cesarean), whether the child was ever breastfed, insurance type (public, private, or unknown/other), whether the child was ever in daycare, whether there was a young sibling in the household, and whether the child had been exposed to household cigarette smoke were *a priori* selected to be added to the model as independent variables. Age was transformed with restricted cubic splines with 4 knots with the *rms*::*rcs* function. Race/ethnicity was categorized as black (non-Hispanic), Hispanic, white (non-Hispanic), and other or unknown. Interquartile odds ratios (aOR) and their 95% CIs were found with *rms*::*summary.rms* and *P* values for each independent variable were calculated with the *rms*::*anova* function. The function *rms*::*vif* was used to check for multicollinearity of independent variables. The *validate* and *calibrate* functions from *rms* over 300 bootstrap replications were used to assess model performance (71).

The range of variance inflation factors for variables other than age obtained with *rms*::*vif* ranged from 1.00 to 1.99 in model 1 and from 1.07 to 2.99 in model 2, indicating low to moderate correlation between the factors, which was not severe enough to warrant corrective measures. Since both linear and nonlinear relationships between age and viral genotype were examined, the linear and nonlinear age variables will be correlated with each other and thus have high variance inflation factors (model 1 range, 23.44 to 184.24; model 2 range, 22.59 to 131.81). However, there was no evidence that age was correlated with any other variables in either model.

The AUC/concordance index (c-index) value was 0.62 in model 1 and 0.74 in model 2. Model performance was assessed with the *validate* and *calibrate* functions in *rms*. After running validate, the corrected Somers’ D rank correlation was 0.06 in model 1 and 0.30 in model 2. After calibration, the mean absolute error rate was 0.05 in both model 1 and model 2.

The association between phenotypes (demographic and clinical information) and the viral phylogenies of RSV-A were also studied by comparative phylogenetic analysis with their metadata. BMCMC phylogenies were inferred from the MrBayes analysis described above. To determine whether the viruses displayed phylogenetic clustering by demographic and clinical characteristics, we grouped the viruses by each metadata category individually (Table 1). The overall statistical significance of association between the phylogenies and host information was determined using two phylogeny-trait association statistic tests, the parsimony score (PS) and the association index (AI) tests, implemented in the Bayesian tip association significance testing (BaTS) program (72). The null hypothesis is that clustering by host and geographic information is not less than that expected by chance. A significance level of *P* < 0.05 was used, and a null distribution of these statistics was determined using the posterior distribution of BMCMC phylogenies.

### 16S rRNA sequencing and data analyses.

Microbiome profiles were available for 168 RSV-A (114 with the G-gene duplication and 54 without the G-gene duplication) and 102 RSV-B complete genome sequences from infants in the INSPIRE cohort from the middle Tennessee region during from seasons 2012 to 2014. We have previously described in detail the methods used to characterize the nasopharyngeal microbiome using nasal washes in infants enrolled in INSPIRE (35, 37). In brief, following bacterial DNA extraction with the Qiagen PowerSoil kit, the V4 region of the 16S rRNA gene was amplified using universal 515F/806R primers to construct the sequencing libraries (73). The libraries were then sequenced on an Illumina MiSeq platform with 2 × 300-bp reads. Negative and positive controls (with known taxonomic composition) were amplified and sequenced concurrently for quality control.

The R package *dada2* (74) was used to process the sequences and construct amplicon sequence variants (ASVs). Taxonomy was assigned with the SILVA database (75). Sequences were subsequently processed with the “prevalence” method in the R package *decontam* (76) to remove suspected contaminants. Prior to statistical analysis, samples with <1,000 reads were removed (*n* = 6). Statistical analysis was performed with MGSAT, which runs in R. The MGSAT pipeline wraps a number of R packages, as described below, to generate figures and perform statistical analysis.

Estimates of microbial richness, alpha diversity, and beta diversity were done with *vegan* (77) at the ASV level. All ASVs, regardless of abundance, were included. To control for differences in sequencing depth, counts were randomly rarefied to the lowest library size (*n* = 2,062), and then each index was computed. For each index, this rarefaction and computation process was repeated (*n* = 400), and the results were averaged. Microbial richness and alpha diversity were assessed using the Hill numbers N0, N1, and N2, which are, respectively, richness, the exponential of the Shannon index, and the inverted Simpson index (78). Generalized linear models were fit to test for significant associations between metadata categories and richness/alpha-diversity indices. The beta diversity was assessed with the Bray-Curtis dissimilarity index computed on simple proportions, and the PERMANOVA test as implemented in *vegan*::*adonis2* was used to test for significant differences between overall microbial composition and metadata groupings. Principle coordinate analysis (PCoA) was performed in *vegan* in order to ordinate the dissimilarity data and plot it in two-dimensional space. PCoA vectors and centroids were extracted with the *vegan*::*betadisper* function. The *vegan*::*betadisper* function in *vegan* was also used to test for differences in variance between the groups.

Prior to all other analyses, we conducted unbiased filtering by eliminating all taxa that were detected on average <10 times, taxa with a minimum quantile mean fraction of <0.25, and taxa with a minimum quantile incidence fraction of <0.25. After this filtering, 33 genera remained for analysis. The absolute counts from the removed taxa were aggregated into a category “other,” which was taken into account when computing simple proportions during data normalization but were otherwise discarded. This was done to remove the penalty associated with multiple comparisons and to remove likely noninformative data. Testing for statistically significant associations between metadata categories and taxa was performed with DESeq2 (79), which uses a Wald test with the Benjamini-Hochberg correction to control for multiple comparisons; adjusted *P* values (*q* values) are reported. Heatmaps to examine broad clustering of microbial taxa were generated with ComplexHeatmap (80) using Bray-Curtis dissimilarities. The number of cluster splits was determined by partitioning around medoids (method pamk in R package *fpc* [81]). Significant associations between cluster splits and metadata categories were tested for with the G-test of independence with Williams’ correction. For all microbiome analyses, significance was considered if the *P* or *q* values were <0.05, as appropriate.

To explicitly test for interactions between RSV genotype and host sex and their association with microbiome parameters, the function *stats*::*glm* in R was used to run generalized linear regression models. The general format of the models was as follows: glm(x ~ RSV_genotype * infant_sex, data=input_data, family=gaussian), where x was a microbiome parameter, and RSV_genotype was either RSV-A versus RSV-B or, among RSV-A only, G duplication incidence. The iterated microbiome response variables were as follows: (i) pseudo-log-transformed taxon relative abundance after calculating simple proportions (1e–6 was added to each proportion to prevent uncalculatable 0 values); (ii) alpha-diversity as assessed with Hill numbers N0, N1, and N2, as described previously; and (iii) the first 10 PCoA axes calculated after ordination from Bray-Curtis dissimilarities. The Benjamini-Hochberg correction was used to adjust for multiple comparisons.

### Data availability.

All 339 sequences generated as part of this study were submitted to GenBank under BioProject IDs PRJNA241108, PRJNA225816, and PRJNA267583 under accession numbers KJ672424.1 to KX894807.1 (KM042382.1, KM042387.1, and KU950597.1 are partial sequences).
