# Supplementary material for: Influence of Sex on Respiratory Syncytial Virus Genotype Infection Frequency and Nasopharyngeal Microbiome
Source: J Virol. 2023 Feb 23;97(3):e01472-22. doi: 10.1128/jvi.01472-22 (PMC10062153; doi:10.1128/jvi.01472-22)
Supplement: Supplemental file 1 — Fig. S1 and Table S1. Download jvi.01472-22-s0001.pdf, PDF file, 1.4 MB [file jvi.01472-22-s0001.pdf]

# SUPPLEMENTAL FIGURE 1

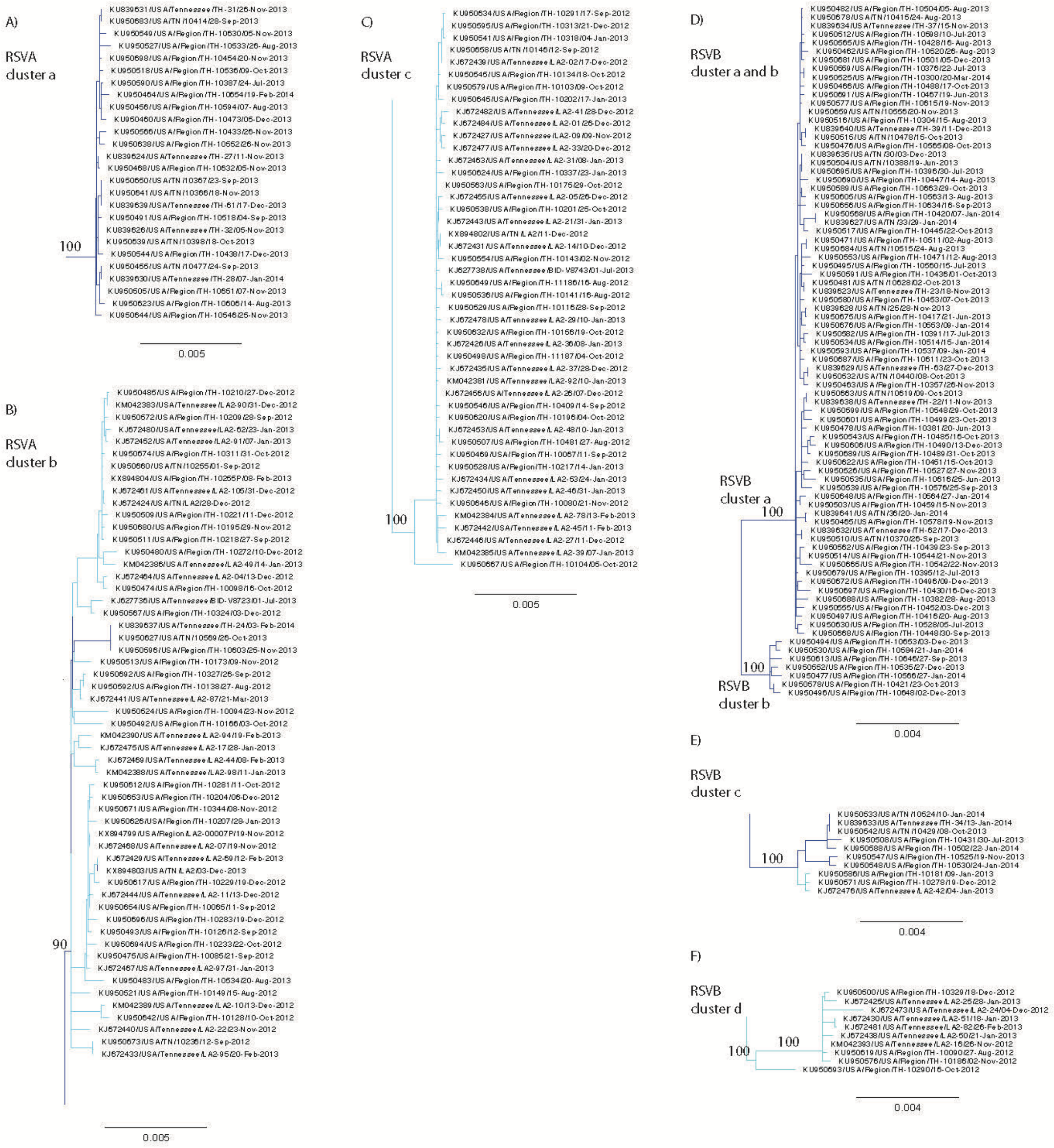

**Supplementary Legends**

**Figure S1.** Magnification of clusters a, b, and c in RSVA and clusters a, b, c, and d in RSVB. Bootstrap values of clusters are indicated, and the scale bars represent genetic distance.

**Table S1.** Country of origin of publicly available and newly generated RSV samples used to construct maximum likelihood phylogenetic trees. For samples collected from the United States, the state the sample was collected from is also listed, where available.

| **Country** | **RSV-A (N=875)** | **RSV-B (N=389)** |
|---|---|---|
| Argentina | 6 | 3 |
| Australia | 2 | 0 |
| Belgium | 2 | 4 |
| China | 5 | 1 |
| Germany | 3 | 2 |
| Hong Kong | 4 | 0 |
| India | 2 | 0 |
| Italy | 5 | 8 |
| Jordan | 58 | 27 |
| Kenya | 45 | 16 |
| Mexico | 5 | 5 |
| Netherlands | 32 | 30 |
| New Zealand | 51 | 41 |
| Peru | 102 | 20 |
| South Africa | 2 | 3 |
| United Kingdom | 0 | 1 |
| United States* | 514 | 212 |
| Colorado | 4 | 0 |
| Maryland | 1 | 0 |
| New York | 109 | 71 |
| Tennessee | 380 | 132 |
| Wisconsin | 20 | 7 |
| Vietnam | 37 | 16 |
| *The state two clinical RSV-B isolates were collected from was not listed in the Genbank record. | | |
